# The Effect of Recurrent Floods on Genetic Composition of Marble Trout Populations

**DOI:** 10.1371/journal.pone.0023822

**Published:** 2011-09-08

**Authors:** José Martin Pujolar, Simone Vincenzi, Lorenzo Zane, Dusan Jesensek, Giulio A. De Leo, Alain J. Crivelli

**Affiliations:** 1 Department of Biology, University of Padova, Padova, Italy; 2 Department of Environmental Sciences, University of Parma, Parma, Italy; 3 Tolmin Angling Association, Most na Soci, Slovenia; 4 Station Biologique de la Tour du Valat, Arles, France; Barnard College, Columbia University, United States of America

## Abstract

A changing global climate can threaten the diversity of species and ecosystems. We explore the consequences of catastrophic disturbances in determining the evolutionary and demographic histories of secluded marble trout populations in Slovenian streams subjected to weather extremes, in particular recurrent flash floods and debris flows causing massive mortalities. Using microsatellite data, a pattern of extreme genetic differentiation was found among populations (global *F*_ST_ of 0.716), which exceeds the highest values reported in freshwater fish. All locations showed low levels of genetic diversity as evidenced by low heterozygosities and a mean of only 2 alleles per locus, with few or no rare alleles. Many loci showed a discontinuous allele distribution, with missing alleles across the allele size range, suggestive of a population contraction. Accordingly, bottleneck episodes were inferred for all samples with a reduction in population size of 3–4 orders of magnitude. The reduced level of genetic diversity observed in all populations implies a strong impact of genetic drift, and suggests that along with limited gene flow, genetic differentiation might have been exacerbated by recurrent mortalities likely caused by flash flood and debris flows. Due to its low evolutionary potential the species might fail to cope with an intensification and altered frequency of flash flood events predicted to occur with climate change.

## Introduction

Climate change poses a serious threat to species persistence. Many species will experience selection in new directions and at new intensities, and the degree to which species respond adaptively to dynamic and uncertain futures will determine their survival over the coming decades and millenia [1]. Intensification of weather extremes and associated catastrophic disturbances is emerging as one of the most important aspects of climate change and research is advancing from studying the impacts of changes in mean climate values (trend effects) to those produced by changes in the magnitude or frequency of extreme events (event effects) [2]. Catastrophes are characterized by their statistical extremeness combined with a short duration relative to the life cycle of the organisms affected; they can disrupt ecosystem, community or population structure and change resources, substrate availability, or the physical environment [2]–[4]. Evidence suggests that the frequency and intensity of extreme weather events (i.e. floods, droughts) is increasing in many regions in response to global climate change [5]–[7]. Despite the urgent need to advance research on extreme events and catastrophic disturbances, their evolutionary consequences have largely been unexplored [8].

Freshwater salmonids are commonly subject to substantial environmental variability in the form of changes in stream flow at different time scales [9]–[11]. Extreme events such as floods, droughts or landslides play an important role in the regulation of population dynamics in salmonids, to the extent that in high-density streams, fish demography and persistence might be largely shaped by extreme flow events [12]–[15]. The direct and short-term effects of floods are largely a result of high-water velocities and sediment movement that cause the displacement and death of fish. The impact of floods is expected to be higher in the coming years. According to IPCC predictions, an increase of rainfall is expected in the next 50 years along with an intensification and altered frequency of catastrophic disturbances [5]. Recent advances in the statistical theory of extreme events suggest that large flood events will be more frequent, with return times markedly shorter than expected even in the absence of climate change [16].

Our model system is the stream-dwelling salmonid marble trout *Salmo marmoratus*, an endemism of the Adriatic Sea and its tributaries, currently restricted to Northern Italy, former Yugoslavia (Slovenia, Croatia, Bosnia-Herzegovina) and Albania [17]. Marble trout is considered to be one of the most endangered species in the Adriatic basin [18]. For decades, massive restocking practices have been conducted throughout its natural range by means of introduction of exotic brown trout. Hybridization between marble and brown trout has been so extensive that hybrids now dominate most rivers [19]. Molecular data confirm a high level of introgression in the Po river in Northern Italy [20], [21], the Soca river system in the Italian/Slovenian border [18], [22], [23], and recently, the Adige river system in South Tyrol [24], [25]. However, all studies also reported pure populations of marble trout in headwaters of all river systems, persisting above natural barriers (i.e. waterfalls). While those barriers prevent the upstream migration of conspecifics and consequent hybridization, the secluded nature and the small size of the remnant marble trout populations makes them extremely vulnerable to stochastic factors, including environmental events such as floods, droughts or landslides.

A project for the conservation and rehabilitation of marble trout in Slovenia started in 1993 [17]. Only seven remnant pure (non-introgressed with brown trout; [23]) populations of marble trout remain in the Adriatic basin of Slovenia, persisting in totally isolated headwaters without predators or fishing [17]. Recurrent major floods and debris flows are the most important risk factor for the viability of marble trout populations in Slovenian streams [15], [26]. The trigger of debris flows in the area is extreme precipitation, with records of intense flows going back to the 18^th^ century, and with a presumable occurrence interval of approximately 50 years (i.e. in the 20^th^ century major floods were recorded in 1926, 1954 and 1990; [27]). However, an intensification of the frequency of flash floods has been observed in the last decade, with four important flood events recorded in the 1999–2010 period (Table 1).

**Table 1:** Occurrence and intensity of flood events in the study area in the period 1999–2010, showing medium floods (m, 10 to 20-year recurrence interval) and major floods (M, 50 to 100-year recurrence interval).

Population	1999	2000	2001	2002	2003	2004	2005	2006	2007	2008	2009	2010
Zadlascica	A/m					A/M			A/M		S/m	A/m
Lipovscek	A/M	A/m			A/m	A/M			A/M		A/M	A/m
Sevnica			A/m			A/M	A/m				S/m	S/m
Studenc		A/m	A/m			A/M	A/m				S/m	S/m

A = Autumn; S = Spring.

**Figure 1:**
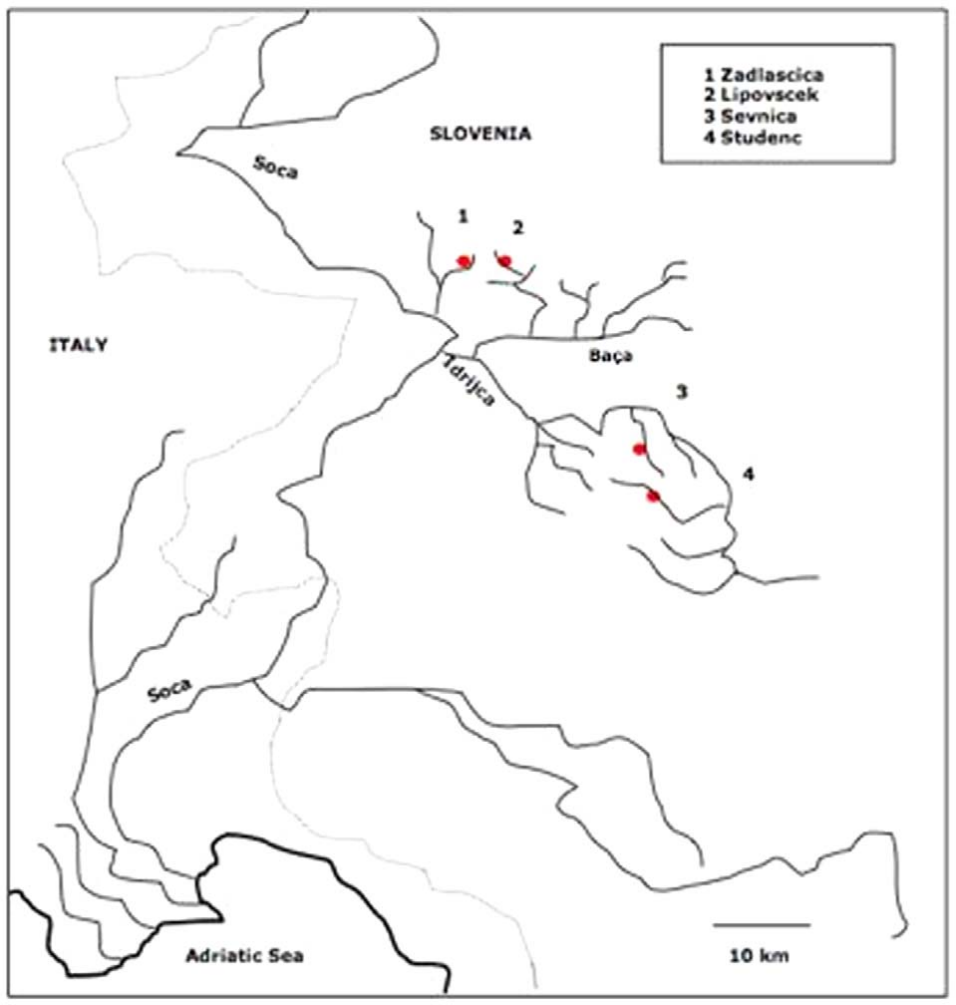
Location of all marble trout sampling locations.

The aim of this study is to explore the impact of catastrophic weather events on the genetic composition of isolated marble trout populations from the Adriatic basin of Slovenia (Figure 1). As a result of intense precipitation and associated flash flood and debris flows, which causes mass mortalities but does not alter connectivity among isolated streams and basins, low levels of genetic diversity and high genetic substructuring at local geographic scale are expected. In this study, we were particularly interested in the comparison of pre- and post-flood samples, testing shifts in genetic composition and genetic diversity and estimating the possible existence of bottleneck/population declines.

## Results

A total of 18 out of 24 loci were polymorphic, showing from 2 to 8 alleles per locus (Table 2). All locations showed low levels of genetic diversity as evidenced by heterozygosities <0.25 and allelic richness <2 (Table 3). Many loci showed a discontinuous allele distribution, with missing alleles across the allele size range, and few or no rare alleles (Table S1). A total of 31 location-specific private alleles were found, representing 49% of the total number of alleles sampled. Across locations, Studenc showed the highest genetic diversity, with intermediate levels found in Zadlascica and the lowest genetic variability found in Lipovscek and Sevnica (Table 3). When comparing pre- and post-flood samples, a moderate drop in genetic diversity was observed in all four locations in terms of *H*_o_ and *H*_e_ and allele richness (AR), mostly due to the loss of rare alleles (Table 3; Table S1). However, statistic comparisons between pre- and post-flood values of genetic diversity were non-significant at all locations: Zadlascica (*H*_o_: p = 0.714; *H*_e_: p = 0.891; AR: p = 0.747), Lipovscek (*H*_o_: p = 0.650; *H*_e_: p = 0.650; AR: p = 0.396), Sevnica (*H*_o_: p = 0.591; *H*_e_: p = 0.554; AR: p = 0.551) and Studenc (*H*_o_: p = 0.726; *H*_e_: p = 0.451; AR: p = 0.999).

**Table 2:** Microsatellite loci analyzed in marble trout including microsatellite type, repeat motif, size range (in bp), maximum number of alleles per locus (NA), and species in which the microsatellites were originally developed (*Salmo salar*, *S. trutta*, *S. marmoratus*).

Name	Type	Motif	Size	NA	MX	Species	Reference
CA048828	EST	CA	246–276	4	1	*S. salar*	Vasemagi *et al.* 2005
CA048687	EST	AC	216	1	2	*S. salar*	Vasemagi *et al.* 2005
CA060208	EST	CA	166	1	2	*S. salar*	Vasemagi *et al.* 2005
CA039543	EST	AT	145–147	2	1	*S. salar*	Vasemagi *et al.* 2005
CB515794	EST	GT	262–264	2	2	*S. salar*	Vasemagi *et al.* 2005
CB512797	EST	AC	375–407	7	2	*S. salar*	Vasemagi *et al.* 2005
CA060177	EST	TGAC	300–320	5	1	*S. salar*	Vasemagi *et al.* 2005
CA769358	EST	AC	98	1	2	*S. salar*	Vasemagi *et al.* 2005
CA053293	EST	AC	156–158	2	1	*S. salar*	Vasemagi *et al.* 2005
CA040282	EST	AT	121	1	2	*S. salar*	Vasemagi *et al.* 2005
CA059136	EST	TA	329–355	8	2	*S. salar*	Vasemagi *et al.* 2005
CA058902	EST	TA	179–181	2	2	*S. salar*	Vasemagi *et al.* 2005
CA050376	EST	GT	283–291	2	1	*S. salar*	Vasemagi *et al.* 2005
BG935488	EST	CAAT	131–143	2	1	*S. salar*	Vasemagi *et al.* 2005
CL47345	EST	TG	220–232	3	1	*S. salar*	Siemon *et al.* 2005
Str73	Genomic	CT	151–165	3	1	*S. trutta*	Estoup *et al.* 1993
Str151	Genomic	GT	216	1	1	*S. trutta*	Estoup *et al.* 1993
Str85	Genomic	CT	171–181	3	2	*S. trutta*	Presa & Guyomard 1996
Str543	Genomic	CT	130–134	3	1	*S. trutta*	Presa & Guyomard 1996
Str591	Genomic	CT	152–156	3	2	*S. trutta*	Presa & Guyomard 1996
T3-13	Genomic	GT	162–168	4	2	*S. trutta*	Estoup *et al.* 1998
Strutta58	Genomic	GT	104–124	4	2	*S. trutta*	Poteaux *et al.* 1999
Ssa85	Genomic	GT	108	1	2	*S. salar*	O'Really *et al.* 1996
BFRO001	Genomic	TG	202–212	3	1	*S. marmoratus*	Snoj *et al.* 1997

Information is also given on the combination of loci used in each multiplex (MX, 1 and 2).

**Table 3:** Summary of genetic variability estimates across samples including number of individuals (N), expected (*H*_e_) and observed (*H*_o_) heterozygosities, total (TNA) and mean (MNA) number of alleles and allelic richness (AR).

Sample	N	*H*_e_	*H*_o_	TNA	MNA	AR
**1A- Zadlascica 2007**	30	0.179	0.166	29	1.56	1.50
**1B- Zadlascica 2008**	15	0.173	0.163	28	1.50	1.49
**2A- Lipovscek 2004**	30	0.116	0.141	30	1.67	1.45
**2B- Lipovscek 2005**	28	0.094	0.112	26	1.44	1.33
**3A- Sevnica 2004**	30	0.128	0.138	32	1.78	1.60
**3B- Sevnica 2005**	30	0.102	0.110	30	1.61	1.43
**4A- Studenc 2004**	30	0.242	0.204	35	1.94	1.75
**4B- Studenc 2005**	30	0.205	0.189	34	1.89	1.72

All loci were at HWE after Bonferroni correction. A neutrality test using LOSITAN suggested no loci to be subject to balancing selection or directional selection. No difference in values of genetic diversity was found between EST-derived microsatellites and genomic microsatellites, including *H*_o_ (EST-microsatellites: 0.126; genomic microsatellites: 0.195; p = 0.232), *H*_e_ (EST-microsatellites: 0.138; genomic microsatellites: 0.180; p = 0.464) and AR (EST-microsatellites: 2.87; genomic microsatellites: 2.78; p = 0.964).

A highly significant extreme overall genetic differentiation was found among samples (*F*_ST_ = 0.716; p<0.001). All pairwise *F*_ST_ and genetic distances between samples from different locations were high (*F*_ST_ = 0.649–0.779; D_CE_ = 0.592–0.839) and statistically significant (p<0.001; Table 4). By contrast, comparison of pre- and post-flood samples from the same location showed low non-significant *F*_ST_ and genetic distances with the exception of Studenc (*F*_ST_ = 0.041, p = 0.020; D_CE_ = 0.021, p = 0.035). Accordingly, comparison of allele frequencies between pre- and post-flood samples using an exact test showed no temporal differences at Lipovscek (p = 0.993), Sevnica (p = 0.450) and Zadlascica (p = 0.985), while significant differences were found at Studenc (p<0.001) caused by loci CA059136 and Str151.

**Table 4:** Pairwise *F*_ST_ estimates (above diagonal) and genetic distances (D_CE_, below diagonal) between samples, labeled as in Table 3.

Sample	1A	1B	2A	2B	3A	3B	4A	4B
**1A**	***	0.001	0.737**	0.758**	0.707**	0.733**	0.673**	0.681**
**1B**	0.004	***	0.752**	0.779**	0.719**	0.753**	0.679**	0.673**
**2A**	0.712**	0.719**	***	0.001	0.738**	0.761**	0.693**	0.717**
**2B**	0.735**	0.743**	0.007	***	0.755**	0.778**	0.709**	0.734**
**3A**	0.658**	0.669**	0.606**	0.601**	***	0.002	0.649**	0.673**
**3B**	0.673**	0.680**	0.598**	0.592**	0.011	***	0.668**	0.694**
**4A**	0.833**	0.839**	0.753**	0.779**	0.630**	0.673**	***	0.040*
**4B**	0.787**	0.793**	0.725**	0.753**	0.595**	0.609**	0.021*	***

*p<0.05;

**p<0.001.

**Figure 2:**
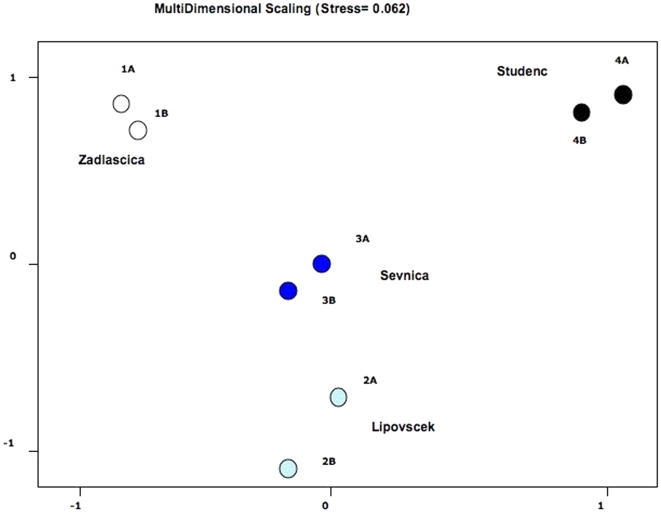
Plots of the values of the first and second principal coordinates obtained from Cavalli-Sforza and Edwards (1967) chord distances at all loci. Samples labeled as in Table 3. A = pre-flood samples; B = post-flood samples. Stress value = 0.062.

Accordingly, an AMOVA analysis partitioned genetic differentiation significantly among locations (*F*_CT_ = 0.713; p<0.001) but not among (pre- and post-flood) samples within locations (*F*_SC_ = 0.011; p = 0.067) (Table 5). We conducted a Multidimensional Scaling analysis using D_CE_ at all loci (Figure 2). When plotting the values of the first and second principal components, samples clustered according to location, the Zadlascica location (from a tributary of the Soca river) appeared very different from the rest of locations (from tributaries of the Idrijca river), but so did Studenc despite being 20 km apart from Sevnica. In this sense, when testing Isolation-by-Distance, no correlation was found between genetic and geographic (shortest waterway) distances (r = 0.715; F = 4.19; p = 0.110). Assignment analysis using STRUCTURE confirmed the extreme genetic differentiation among locations and suggested a scenario with K = 4 groups as the most likely (p<0.001), corresponding to the four sampled locations, Zadlascica, Lipovscek, Sevnica and Studenc.

**Table 5:** Results from AMOVA analysis partitioning genetic differentiation among locations (4 locations: Zadlascica, Lipovscek, Sevnica and Studenc) and among samples within locations (pre- and post-flood samples).

AMOVA	Sum of squares	Var	*F*
**Among locations**	1124.41	3.46	*F*_CT_ = 0.713 (p = 0.000)
**Among samples within locations**	8.88	0.02	*F*_SC_ = 0.011 (p = 0.067)
**Within samples**	570.76	1.38	
**Overall**	1704.06	4.86	*F*_ST_ = 0.716 (p = 0.000)

**Figure 3:**
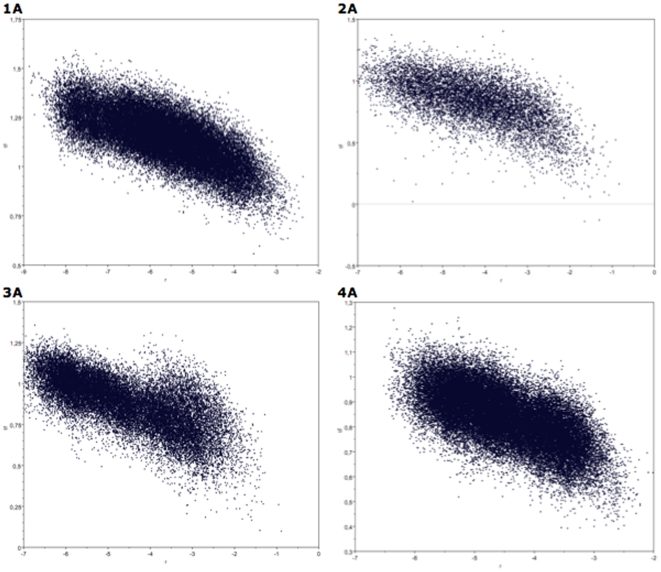
Plots of the simulated points from the marginal posterior distributions of log_10_ (*r*) (x-axis) and log_10_ (t_f_) (y-axis). Samples labeled as in Table 3.

The MSVAR procedure for assessing past demographic history strongly supported a decline in all marble trout populations. All sampled points of log_10_(*r*) were substantially below zero in all eight samples, suggesting that the past population size was larger than the current population size (Figure 3). All sampled points of log_10_(θ) were negative, pointing to small current effective population sizes. Using a conservative mutation rate of μ = 10^−4^, *N*_1_ varied across locations between 3 and 41 individuals, with a maximum upper-bound CI of *N*_1_ of 396 individuals (Table 6). Using higher mutation rates (i.e. 5×10^−4^ and 10^−3^), *N*_1_ values <than 1 were obtained, while lower mutation rates (i.e. 5×10^−5^) yielded unrealistic *N*_0_ values. The time of the bottleneck varied between 31 and 268 generations across locations (or roughly 100–800 years assuming a generation time of 2.7–3 years), with a maximum upper-bound CI of 2300 generations (roughly 7000 years).

**Table 6:** Summary statistics of MSVAR across all locations, including the genetic parameter θ = 2*N*_0_μ, which was calculated using a mutation rate of μ = 10^−4^, *r* = *N*_0_/*N*_1_ (*N*_0_ = current effective population size; *N*_1_ = effective population size at the time of population expansion or decline), and *t*_f_ = *t*_a_/*N*_0_ (*t*_a_ = number of generations since the beginning of the expansion/decline).

	1A	1B	2A	2B	3A	3B	4A	4B
**θ**	0.0006	0.0024	0.0006	0.0063	0.0069	0.0082	0.0014	0.0023
***r***	3.8×10^−5^	1.1×10^−4^	9.0×10^−4^	9.2×10^−4^	6.1×10^−4^	1.1×10^−3^	1.6×10^−4^	1.1×10^−4^
***t*** **_f_**	14.52	11.27	7.67	7.43	8.18	6.47	7.13	8.44
***N*** **_0_**	3	12	31	32	35	41	7	12
	(1–27)	(1–66)	(1–257)	(1–259)	(1–299)	(1–396)	(1–55)	(1–68)
***N*** **_1_**	8.0×10^5^	1.5×10^5^	8.9×10^4^	7.6×10^4^	1.1×10^5^	4.1×10^4^	6.0×10^4^	1.1×10^5^
	(5.6×10^4^–1×10^6^)	(3.6×10^4^–4.3×10^5^)	(8×10^3^–4.7×10^5^)	(8×10^3^–3.7×10^5^)	(1.6×10^4^–4.3×10^5^)	(5.4×10^3^–1.7×10^5^)	(1.7×10^4^–1.7×10^5^)	(2.4×10^4^–3×10^5^)
***t*** **_a_**	31	106	172	179	215	268	83	73
	(1–264)	(1–529)	(1–1400)	(1–1507)	(1–1902)	(1–2300)	(1–301)	(1–405)

95% confidence interval in parenthesis. Samples labelled as in Table 3.

Bottleneck-detection tests (Table 7) using the sofware BOTTLENECK and M_P_VAL showed some indications of population contraction at all locations. Garza and Williamson [28] suggested that values of M lower than 0.7 would indicate evidence of a bottleneck, while values above 0.8 would denote no bottleneck history. In our data set, all Zadlascica and Studenc samples plus the pre-flood Lipovscek sample showed M values between 0.518–0.661. The two Sevnica samples plus the post-flood Lipovscek sample showed M values between the 0.7–0.8 limits.

**Table 7:** Results of the bottleneck analyses.

Sample	P value (Wilcoxon test)	M value (M_P_VAL)	p value (M_P_VAL)
**1A**	0.009	0.566	0.001
**1B**	0.043	0.518	0.001
**2A**	0.103	0.661	0.038
**2B**	0.381	0.733	0.343
**3A**	0.691	0.744	0.328
**3B**	0.606	0.793	0.751
**4A**	0.044	0.638	0.011
**4B**	0.193	0.610	0.003

BOTTLENECK: p-value of the one-tail Wilcoxon test for heterozygote excess. M_P_VAL: M is the observed average ratio between number of alleles (k) and range in allele size (r) across loci. Samples labelled as in Table 3.

## Discussion

### Extreme differentiation among marble trout populations

Freshwater fish species show a greater average degree of genetic differentiation among locations than marine species, resulting from the isolation of fish populations among drainages [29]. The marked zoogeography produced by the historical patterns of isolation among drainages is subjected to the continuous remodeling of the river drainage and to climatic fluctuations of which the glacial/interglacial periods of the Pleistocene played a crucial role [30]. Species respond actively to fluctuations in their natural range, with hydrographic networks being used for colonization and for retreat throughout geological times. Higher levels of population subdivision have been found in many salmonid studies reflecting complex genetic structures often resulting from isolation among drainages. In brook charr *Salvelinus fontinalis*, a global *F*_ST_ of 0.37 was found among 26 populations from La Maurice National Park in Canada located 3–42 km apart [31]. In cutthroat trout *Oncorhynchus clarkii*, Taylor *et al.* [32] found an overall *F*_ST_ of 0.32 and pairwise *F*_ST_ values up to 0.45 in populations from British Columbia, while the recent study of Pritchard *et al.* [33] reported a global *F*_ST_ of 0.41 for Rio Grande populations. In bull trout *Salvelinus confluentus*, Taylor and Costello [34] found an *F*_ST_ = 0.33 among 20 North-West America coastal locations, while recent papers reported an overall *F*_ST_ of 0.15 and pairwise *F*_ST_ values up to 0.31 in Alberta [35] and pairwise *F*_ST_ values up to 0.66 between lake samples in a 50 km area off Montana [36].

Collectively, the extreme level of genetic differentiation found among Slovenian marble trout populations (overall *F*_ST_ = 0.716; pairwise *F*_ST_ between drainages = 0.649–0.779; 49% private alleles sampled) exceeds the highest values reported in salmonid populations found above waterfalls and/or in small streams like the ones in this study. Such extremely high *F*_ST_ is indicative of complete genetic isolation that has persisted over time. This is concordant with the previous study of Fumagalli *et al.* [23], which reported a global *F*_ST_ of 0.66 in the same area using a smaller number of microsatellites. At present, the four locations in our study are completely isolated from each other and from the main river drainage by means of impassable natural barriers (waterfalls) that restrict dispersal abilities. Baloux and Lugon-Moulin [37] argued that even in the total absence of gene flow, extreme genetic differentiation of the magnitude found in our study is not expected due to the high mutation rate of microsatellites [38], with appearance of new variants counteracting within-population fixation of alleles. However, the low level of genetic diversity observed in all locations (low heterozygosities and low number of alleles) implies a strong impact of local genetic drift, and suggests that, together with absence of gene flow, genetic differentiation among locations may have been exacerbated by recurrent mortalities likely caused by flash flood and debris flows. The combined effects of genetic drift and inbreeding following demographic bottlenecks may have thus contributed to alter distinctly the genetic composition of each population, so that over time different populations ended up having different alleles at each locus as well as different allele combinations from multiple loci, resulting in the observed pattern of extreme genetic differentiation.

The MSVAR procedure clearly suggested a population contraction with a reduction in population size of 3–4 orders of magnitude to current effective densities of around 3–41 (CI: 1–396) individuals per location. Bottleneck signatures were observed in all samples. First, most polymorphic loci presented a biallelic pattern with few (or none) rare alleles. Second, a discontinuous allele range was found at many loci (Table S1). For instance, only alleles 104, 110, 118 and 124 were sampled across locations at locus Strutta58, which suggests that the non-observed alleles have been lost over time. By contrast, samples collected in tributaries of the river Po in Northern Italy showed a continuous allele distribution and a higher number of alleles at five microsatellite loci [39], suggestive of demographically stable populations with no signatures of bottlenecks. Loci Ssa85 and Str15, which were monomorphic in Slovenian locations (our study), showed 6–9 alleles in Italian locations. Loci Str85, Str543 and Str591, which presented three alleles each in our study, showed higher allelic richness in the Italian samples (7–17 alleles). Pujolar *et al.* [39] also reported a lower genetic differentiation among Italian pure marble trout locations (global *F*_ST_ of 0.235). The different pattern suggested for Italian and Slovenian populations of marble trout, with signatures of a bottleneck only observed in the latter, might be due to the particular geography and the extreme effect of meteorological events in the region of Slovenia studied. Rainfall data have been acquired since 1961 (ARSO, Environmental Agency of Slovenia). While the annual mean precipitation was 2,400 mm in the 1961–2004 period, showing little variation across years, monthly rainfall showed up to 500-fold variation. Morphological features of the streams and watersheds in the region (i.e. high slopes, narrow walls, stream bed fragmentation, limited flood plain) are largely responsible for the high water flow in the stream after heavy rainfalls, and might explain the massive mortalities caused by flood events.

In contrast with the extreme differentiation found at microsatellite loci, one single mitochondrial DNA control region haplotype (MA1) has been reported for marble trout in Slovenia [22], [39]. This suggests that despite the current complete geographic isolation of marble trout populations in Slovenian streams, those populations were interconnected in a relatively recent past prior to the fragmentation of habitats. On the basis of microsatellite data, Fumagalli *et al.* [23] suggested that the pure populations within the Idrijca drainage belonged to an independent river system. Nevertheless, our analysis on a larger number of microsatellite loci does not support the division by river basins, as the two locations from the Idrijca basin in our sampling (Sevnica and Studenc) did not cluster together in the Multidimensional Scaling analysis, and appeared clearly differentiated despite being merely 20 km apart within the same river basin.

### Genetic consequences of serial bottlenecks

The analysis of pre- and post-flood samples in our study allowed us to explore the genetic consequences of population bottlenecks. The effects of bottlenecks are directly related to the increase of stochastic events associated with small population sizes, leading in most cases to a loss in genetic variability [40]. Recently, Bouzat [41] suggested that the potential genetic outcomes of demographic bottlenecks can only be assessed when considering replicated bottlenecked populations. In our study, we explored four separate locations that experienced episodes of massive mortalities with consequent reductions in population size ranging from 56 to 77%. Collectively, and taking into the consideration the magnitude of the demographic decline, only a limited genetic effect was observed. A moderate drop in genetic diversity was found in all locations, both in terms of heterozygosities and allelic richness, but all comparisons were statistically not significant. However, a total of 9 alleles were lost locally, in all the cases representing rare alleles with frequencies <0.05 prior to the flood that were no longer sampled after the flood events. Moreover, bottlenecks resulted in alteration of allele frequencies, particularly secondary alleles, which in some cases experienced a drop of 20–30% in frequency. This was notably observed at Studenc, where genetic composition was significantly different in pre- and post-flood samples, but not at the other populations.

The moderate reduction of allelic diversity and heterozygosity might be attributable to the particular demographic history of those populations. In his recent review, Bouzat [41] emphasized the potential role of population history in determining the outcome of demographic bottlenecks. Specifically, one can expect that populations experiencing recurrent bottlenecks might have had their genetic pool already eroded over time, which would decrease the effectiveness of both purifying selection and random allele elimination. This holds particularly true for the Slovenian marble trout populations in our study, which have been repeatedly impacted by severe flood events, with a presumable occurrence interval of 50–100 years [27]. Bayesian demographic analysis using the MSVAR procedure is concordant with a scenario of serial bottleneck episodes that might have been occurring for 150–1340 years.

On the question of how do the Slovenian population still maintain some polymorphism despite experiencing flood events for hundreds of years, one possible explanation is that our sampling is not representative of the entire population, and other compartments of the population might keep some genetic variation. One hypothesis could be that young fish (age 0+ and 1+) might be less sensible to flows because usually they are not found in the main stream but in more protected small tributaries, repopulating the stream even if all adults have been removed. However, field observations suggest that eggs and young fish are more vulnerable to floods than adults and that there is no recruitment at all when floods occur after reproduction (Crivelli, unpublished information). Alternatively, an upstream part of the population could be preserved. This hypothesis is supported by tagging data (Crivelli, unpublished information) that show non-tagged individuals in post-flood samples, whereas all pre-flood samples had been tagged, which suggests that the newly-arrived non-tagged individuals came from a compartment of the population located upstream, flushed by the flood.

### Coping with environmental change

Our findings strongly suggest that the evolutionary and demographic history of marble trout populations living in secluded Slovenian streams has been shaped by massive mortalities caused by disturbance factors such as flash flood and debris flows. Overall, genetic analysis revealed an extremely high genetic differentiation among remnant pure populations, together with much low levels of genetic diversity in all populations. Effective population sizes estimated using the MSVAR procedure ranged from 3 to 41 (CI:1–396) individuals per population. The low genetic variability found does not seem to affect the viability of the populations so far, as they have persisted up to the present despite recurrent and unpredictable disturbances. To mitigate their ecological impact, fish populations might exhibit several adaptations that result from trade-off among growth, reproduction and survival. According to life-history theory, recurrent floods can have important evolutionary consequences by selecting for life-histories that are synchronized to either avoid or exploit the direct and indirect effects of extreme flows [42]. However, due to the predicted intensification of extreme weather leading to increased floods, heatwaves, droughts and rainfall in the next 50 years [5], [43], marble trout may not have the sufficient genetic potential for the evolution of life-histories able to mitigate their impact. Recent studies by the European Commission's Joint Research Centre (JCR) have confirmed rising temperatures (around 1.5°C in the last 35 years) and higher levels of precipitation in Slovenia, with a projected increase in the occurrence of extreme rainfall events and flash floods. For 2020, Lehner *et al.* [44] predict major flood events for the Adriatic basin of Slovenia to become more frequent and intense. This might jeopardize the survival of marble trout as the occurrence of consecutive floods in a very short period of time could wipe out the population. This nearly occurred in the Lipovscek stream: a good recovery was observed following the 2004 flood, but in September 2007 a new important debris flood occurred with a mortality of 92.4% that left only 38 fish in the whole population. After a new major flood in 2009 followed by a moderate flood in 2010, the population has been decimated to only 10 individuals, so that at present, the recovery of Lipovscek remains uncertain (Crivelli, unpublished information). Medium flows (10 to 20-year recurrence interval) have also become more frequent with 3–5 moderate flow events observed in the last 10 years in the four populations in our study, and additionally, since 2009 spring floods have been observed for the first time in the region.

Collectively, while the Slovenian marble trout populations have coped successfully with recurrent yet unpredictable disturbance events over time, the low genetic variation found in the species makes it difficult to assess its evolutionary potential since a genetically depauperate population might fail to adapt to future environmental change. Moreover, all populations are closed, and there is no potential for spontaneous colonization of new habitats or re-colonization after local extinction. It has been proposed that an effective population size of 500 individuals is large enough to maintain genetic diversity for key history traits [45]. The estimation in our study of effective population sizes of 3–41 individuals is one-two orders of magnitude lower than the threshold of 500 individuals, plus the upper-bound CI of population size does not overlap with the threshold value. This suggests that the Slovenian marble trout populations are beyond the critical population size for genetically secure populations. The future development of an integrated genetic/ecologic model that explores different demographic scenarios with varying degree of intensity and frequency of flood events might help disentangling the role of catastrophic disturbances in determining the genetic structure and genetic diversity of marble trout.

## Materials and Methods

### Sampling

All animal work was approved by the Ministry of Agriculture, Forestry and Food of Republic of Slovenia and the Fisheries Research Institute of Slovenia. Original title of the Plan: RIBISKO - GOJITVENI NACRT za TOLMINSKI RIBISKI OKOLIS, razen Soce s pritoki od izvira do mosta v Cezsoco in Krnskega jezera, za obdobje 2006–2010. Sampling was supervised by the Tolmin Angling Association (Slovenia).

A total of 223 pure marble trout *Salmo marmoratus* individuals were caught using electrofishing at four tributaries of the Soca (1.Zadlascica), Baca (2.Lipovscek) and Idrijca (3.Sevnica and 4.Studenc) rivers in Slovenia (Figure 1). During field work fin clips were collected from anaesthetised individuals that were tagged and immediately released back into the streams. At Lipovscek, Sevnica and Studenc, samples were obtained in September 2004 prior to a flooding event that caused a mortality of 56.4%, 77.6%, and 67.9% in fish ≥ age-1, respectively. Post-flood samples were obtained at all three locations in September 2005. At Zadlascica, samples were obtained in September 2007 prior to a flooding event that caused a 74.9% drop in population size. Post-flood samples from Zadlascica were obtained in September 2008. Number of individuals in post-flood samples were constrained to the high mortalities and consequent low number of survivors, with only 15 individuals left in the case of Zadlascica after the flash flood event.

### Microsatellite amplification

Minute sections of tissue from ethanol-preserved finclips were digested in a lysis buffer containing 100 µl TE Buffer, 7 µl 1 M DTT (dithiothreitol) solution pH 5.2 (diluted in 0.08 M NaAC) and 2 µl proteinase K solution (20 mg/ml) for at least 8 hours at 56°C. After incubation at 96°C for 10 min, samples were centrifuged at 13,000 rpm for 11 min, and the supernatant was stored at −20°C.

All samples were scored for a total of 24 microsatellite loci (Table 2). 15 loci were derived from Expressed Sequence Tags (ESTs) previously described by Vasemagi *et al.* [46] and Siemon *et al.* [47] in Atlantic salmon *Salmo salar*. We selected those loci with the highest genetic variation and a positive cross-species amplification in brown trout *Salmo trutta*. Additionally, 9 genomic microsatellite loci isolated and characterized from other salmonids, seven from brown trout (Str73, Str151: [48]; Str85, Str543, Str491: [49]; T3-13: [50]; Strutta58: [51]), one from Atlantic salmon (Ssa85: [52]) and one from marble trout (BFRO001: [53]) were amplified and scored. Microsatellites were grouped in two separate multiplexes in order to reduce polymerase chain reaction (PCR) and genotyping costs (Table 2).

PCR products were obtained in a GeneAmp PCR System 2700 Thermocycler (Applied Biosystems) using the QIAGEN Multiplex PCR Kit. PCR reactions consisted of 2 µl template DNA, 5 µl QIAGEN Multiplex PCR Master Mix, 0.2 µl 10 µM forward and reverse primers, and water up to 10 µl. PCR conditions were as follows: 3 min at 95°C, 35 cycles of 30 sec at 94°C, 90 sec at 57°C and 1 min at 72°C, and final elongation for 5 min at 60°C. PCR products were visualized in 1.8% agarose gels and screened for microsatellite polymorphism using an ABI 3130 AVANT automatic capillary sequencer (Applied Biosystems). Alleles were sized according to a Liz500 (50–500 bp) marker.

### Data analysis

Within-sample genetic diversity statistics were assessed by observed (*H*_o_) and expected (*H*_e_) heterozygosities per locus using GENETIX version 4.05 [54] and allelic richness (AR) using FSTAT [55]. Differences in genetic diversity among samples were tested by one-way ANOVA using STATISTICA version 6.0 (StatSoft Inc.). Deviations from Hardy-Weinberg Equilibrium (HWE), linkage disequilibrium and differences in allele and genotype frequencies among samples were tested using GENEPOP version 3.4 [56].

Neutrality of the markers was tested using the software LOSITAN [57], which implements a *F*_ST_ outlier detection approach. This method evaluates the relationship between *F*_ST_ and expected heterozygosity (*H*_e_), describing the expected distribution of Wright's inbreeding coefficient *F*_ST_ vs. *H*_e_ under an island model of migration with neutral markers. This distribution is used to identify outlier loci that show excessive high or low *F*_ST_ compared to neutral expectations. Such outlier loci are candidates for being subject to selection. We used the 0.99 criterion in order to minimize false positives as suggested by the authors.

Population structure was explored using non-hierarchical and hierarchical *F*-Statistics [58] calculated using ARLEQUIN [59]. First, overall and pairwise *F*_ST_ values were calculated. Genetic differentiation was also partitioned among locations (4 locations: Zadlascica, Lipovscek, Sevnica and Studenc) and among samples within locations (pre- and post-flood samples). Significance tests were assessed with 10,000 permutation tests. In all cases, significance levels were corrected for multiple comparisons using Bonferroni [60]. Pairwise multilocus comparisons between samples were calculated by Cavalli-Sforza and Edwards [61] chord distance (D_CE_) and graphically represented by Multidimensional Scaling (MDS) analysis using STATISTICA version 7.0 (StatSoft). Isolation-by-Distance (IBD) was tested using a Mantel test implemented in GENETIX, by correlating linearized genetic distance (*F*_ST_/(1−*F*_ST_)) vs. geographic distance (shortest waterway distance measured along the streams between pairs of samples).

A model-based clustering algorithm as implemented in the software STRUCTURE [62] was used in order to infer the most likely number of populations in the data. The software organizes individuals into a predefined number of clusters (K) with a given likelihood, which may represent putative groups. The analysis was performed with 1<K<8 to account for population substructuring within species, using the admixture model and without a population prior. The most likely K was determined using the criterion of Evanno *et al.* [63] and then used to assign each individual. A burn-in length of 10^5^ iterations followed by 10^6^ additional Markov Chain Monte Carlo (MCMC) iterations were performed. A minimum of 5 runs were performed for each K to check repeatability of results.

Historical demographic changes were inferred using the Bayesian coalescent-based approach implemented in MSVAR [64]. Using a strict stepwise mutation model, the procedure provides distributions of the exponential growth rate *r* = *N*_0_/*N*_1_ (where *N*_0_ is the present effective population size, *N*_1_ is the effective population size at the time of population expansion or decline), the time since the population started to expand or decline *t*_f_ = *t*_a_/*N*_0_ (where *t*_a_ is the number of generations since the beginning of the expansion/decline) and the genetic parameter θ = 2*N*_0_μ. A mutation rate of μ = 10^−4^ was used [65], [66]. Rectangular priors were chosen for all parameters, with limits of (−9, +5) for log_10_(*r*), log_10_(*t*_f_) and log_10_(θ) and starting values of 1 for θ at each locus, *r* and *t*_f_. The analysis was performed for an exponential model of population change. We used 50,000 thinned updates and a thinning interval of 50,000 steps, with an initial 10% discarded as burn-in. Convergence was assessed using Tracer v1.4 [67].

Finally, we tested for a recent genetic bottleneck episode with two different approaches. First, we used the software BOTTLENECK [68], based on the principle that after a recent reduction of effective population size, number of alleles (k) decreases faster than heterozygosity (*H*_e_) at polymorphic loci. Thus, in a recently bottlenecked population, the observed gene diversity is higher than the expected equilibrium gene diversity (*H*_eq_) which is computed from the observed number of alleles (k), under the assumption of a constant-size (equilibrium) population [69]. We used the Multiple-step Stepwise (TPM) model [70], which consists of mostly one-step mutations but a small percentage of multi-step changes, and is the recommended model for microsatellite data sets rather than the Infinite Alleles (IAM) or Single-step Stepwise (SMM) models [71]. The proportion of singlestep mutation events was set to 90% (variance = 12%). Observed and expected heterozygosities were compared using a Wilcoxon sign-rank test as suggested by Piry *et al.* [68]. Second, we calculated M, the mean ratio between number of alleles (k) and range in allele size (r), assuming that during a bottleneck episode k decreases faster than r (M_P_Val; [28]). Hence, the value of M decreases when a population is reduced in size. Average M was calculated across loci and compared with the critical value M_crit_ estimated after 10,000 simulations and assuming the population to be at equilibrium. In all simulations, three different values of θ were used (5, 10 and 20). A range of mutation models were examined and conservative values were used for p_s_ (frequency of one-step mutations) and Δ_g_ (average size of non one-step mutations), p_s_ = 0.90 and Δ_g_ = 3.5, respectively.

## Supporting Information

Table S1 Allele frequencies at all loci. Samples labelled as in Table 3.
(DOC)
