# Supplementary material for: The Effect of Recurrent Floods on Genetic Composition of Marble Trout Populations
Source: PLoS One. 2011 Sep 8;6(9):e23822. doi: 10.1371/journal.pone.0023822 (PMC3169565; doi:10.1371/journal.pone.0023822)
Supplement: Table S1 — Allele frequencies at all loci. Samples labelled as in Table 3. (DOC) [file pone.0023822.s001.doc]

**Table S1.**

| **Loci** | **1A** | **1B** | **2A** | **2B** | **3A** | **3B** | **4A** | **4B** |
| --- | --- | --- | --- | --- | --- | --- | --- | --- |
| CA048828 |  |  |  |  |  |  |  |  |
| 246 | 0.140 | 0.100 | 0.000 | 0.000 | 0.000 | 0.000 | 0.000 | 0.000 |
| 250 | 0.860 | 0.900 | 0.983 | 1.000 | 1.000 | 1.000 | 0.000 | 0.000 |
| 254 | 0.000 | 0.000 | 0.000 | 0.000 | 0.000 | 0.000 | 1.000 | 1.000 |
| 276 | 0.000 | 0.000 | 0.017 | 0.000 | 0.000 | 0.000 | 0.000 | 0.000 |
| CA039543 |  |  |  |  |  |  |  |  |
| 145 | 1.000 | 1.000 | 0.983 | 1.000 | 1.000 | 1.000 | 1.000 | 1.000 |
| 147 | 0.000 | 0.000 | 0.017 | 0.000 | 0.000 | 0.000 | 0.000 | 0.000 |
| CB515794 |  |  |  |  |  |  |  |  |
| 262 | 0.000 | 0.000 | 0.964 | 1.000 | 0.966 | 0.983 | 0.333 | 0.250 |
| 264 | 1.000 | 1.000 | 0.046 | 0.000 | 0.035 | 0.017 | 0.667 | 0.750 |
| CB512797 |  |  |  |  |  |  |  |  |
| 379 | 0.000 | 0.000 | 0.914 | 0.946 | 0.000 | 0.000 | 0.000 | 0.000 |
| 385 | 0.000 | 0.000 | 0.086 | 0.054 | 0.000 | 0.000 | 0.783 | 0.707 |
| 387 | 0.615 | 0.444 | 0.000 | 0.000 | 0.000 | 0.000 | 0.000 | 0.000 |
| 399 | 0.000 | 0.000 | 0.000 | 0.000 | 0.920 | 1.000 | 0.000 | 0.000 |
| 403 | 0.385 | 0.556 | 0.000 | 0.000 | 0.000 | 0.000 | 0.000 | 0.000 |
| 405 | 0.000 | 0.000 | 0.000 | 0.000 | 0.000 | 0.000 | 0.017 | 0.017 |
| 407 | 0.000 | 0.000 | 0.000 | 0.000 | 0.080 | 0.000 | 0.200 | 0.276 |
| CA060177 |  |  |  |  |  |  |  |  |
| 300 | 0.000 | 0.000 | 0.000 | 0.000 | 0.000 | 0.000 | 0.617 | 0.370 |
| 308 | 0.000 | 0.000 | 0.000 | 0.000 | 0.083 | 0.104 | 0.000 | 0.000 |
| 312 | 0.000 | 0.000 | 0.000 | 0.000 | 0.034 | 0.017 | 0.000 | 0.000 |
| 316 | 0.000 | 0.000 | 1.000 | 1.000 | 0.000 | 0.000 | 0.000 | 0.000 |
| 320 | 1.000 | 1.000 | 0.000 | 0.000 | 0.883 | 0.879 | 0.383 | 0.630 |
| CA053293 |  |  |  |  |  |  |  |  |
| 156 | 0.000 | 0.000 | 0.000 | 0.000 | 0.000 | 0.000 | 1.000 | 1.000 |
| 158 | 1.000 | 1.000 | 1.000 | 1.000 | 1.000 | 1.000 | 0.000 | 0.000 |
| CA059136 |  |  |  |  |  |  |  |  |
| 329 | 1.000 | 1.000 | 0.000 | 0.000 | 0.000 | 0.000 | 0.000 | 0.000 |
| 331 | 0.000 | 0.000 | 0.017 | 0.000 | 0.000 | 0.000 | 0.117 | 0.074 |
| 337 | 0.000 | 0.000 | 0.883 | 0.946 | 0.707 | 0.793 | 0.033 | 0.148 |
| 339 | 0.000 | 0.000 | 0.067 | 0.054 | 0.155 | 0.172 | 0.000 | 0.000 |
| 341 | 0.000 | 0.000 | 0.000 | 0.000 | 0.017 | 0.000 | 0.000 | 0.000 |
| 343 | 0.000 | 0.000 | 0.000 | 0.000 | 0.121 | 0.035 | 0.000 | 0.000 |
| 349 | 0.000 | 0.000 | 0.033 | 0.000 | 0.000 | 0.000 | 0.633 | 0.741 |
| 355 | 0.000 | 0.000 | 0.000 | 0.000 | 0.000 | 0.000 | 0.217 | 0.037 |
| CA058902 |  |  |  |  |  |  |  |  |
| 179 | 0.792 | 0.767 | 1.000 | 1.000 | 0.741 | 0.667 | 0.775 | 0.889 |
| 181 | 0.208 | 0.233 | 0.000 | 0.000 | 0.259 | 0.333 | 0.225 | 0.111 |
| CA050376 |  |  |  |  |  |  |  |  |
| 283 | 0.000 | 0.000 | 0.067 | 0.053 | 0.000 | 0.000 | 0.000 | 0.000 |
| 291 | 1.000 | 1.000 | 0.933 | 0.947 | 1.000 | 1.000 | 1.000 | 1.000 |
| BG935488 |  |  |  |  |  |  |  |  |
| 131 | 0.537 | 0.700 | 1.000 | 1.000 | 0.911 | 1.000 | 1.000 | 1.000 |
| 143 | 0.463 | 0.300 | 0.000 | 0.000 | 0.089 | 0.000 | 0.000 | 0.000 |
| CL47345 |  |  |  |  |  |  |  |  |
| 200 | 1.000 | 1.000 | 1.000 | 1.000 | 0.107 | 0.155 | 0.593 | 0.804 |
| 228 | 0.000 | 0.000 | 0.000 | 0.000 | 0.714 | 0.690 | 0.407 | 0.196 |
| 232 | 0.000 | 0.000 | 0.000 | 0.000 | 0.179 | 0.155 | 0.000 | 0.000 |
| Str73 |  |  |  |  |  |  |  |  |
| 151 | 1.000 | 1.000 | 0.000 | 0.000 | 0.000 | 0.000 | 0.000 | 0.000 |
| 153 | 0.000 | 0.000 | 1.000 | 1.000 | 0.000 | 0.000 | 0.000 | 0.000 |
| 163 | 0.000 | 0.000 | 0.000 | 0.000 | 1.000 | 1.000 | 1.000 | 1.000 |
| Str85 |  |  |  |  |  |  |  |  |
| 171 | 0.259 | 0.233 | 0.565 | 0.571 | 0.173 | 0.125 | 0.614 | 0.560 |
| 179 | 0.741 | 0.767 | 0.065 | 0.036 | 0.827 | 0.875 | 0.204 | 0.280 |
| 181 | 0.000 | 0.000 | 0.370 | 0.393 | 0.000 | 0.000 | 0.182 | 0.160 |
| Str543 |  |  |  |  |  |  |  |  |
| 130 | 0.983 | 1.000 | 0.000 | 0.000 | 0.000 | 0.000 | 0.000 | 0.000 |
| 132 | 0.000 | 0.000 | 0.483 | 0.396 | 0.035 | 0.038 | 0.017 | 0.017 |
| 134 | 0.017 | 0.000 | 0.517 | 0.604 | 0.965 | 0.962 | 0.983 | 0.983 |
| Str591 |  |  |  |  |  |  |  |  |
| 152 | 0.000 | 0.000 | 0.000 | 0.000 | 0.000 | 0.000 | 0.045 | 0.000 |
| 154 | 0.148 | 0.133 | 0.700 | 0.768 | 1.000 | 1.000 | 0.955 | 1.000 |
| 156 | 0.852 | 0.867 | 0.300 | 0.332 | 0.000 | 0.000 | 0.000 | 0.000 |
| T313 |  |  |  |  |  |  |  |  |
| 162 | 0.268 | 0.367 | 0.000 | 0.000 | 0.000 | 0.000 | 0.000 | 0.000 |
| 164 | 0.000 | 0.000 | 0.000 | 0.000 | 0.033 | 0.017 | 0.446 | 0.500 |
| 166 | 0.732 | 0.633 | 1.000 | 1.000 | 0.967 | 0.983 | 0.000 | 0.000 |
| 168 | 0.000 | 0.000 | 0.000 | 0.000 | 0.000 | 0.000 | 0.554 | 0.500 |
| Strutta58 |  |  |  |  |  |  |  |  |
| 104 | 0.933 | 0.933 | 0.000 | 0.000 | 1.000 | 1.000 | 0.569 | 0.776 |
| 110 | 0.000 | 0.000 | 0.000 | 0.000 | 0.000 | 0.000 | 0.103 | 0.155 |
| 118 | 0.067 | 0.067 | 1.000 | 1.000 | 0.000 | 0.000 | 0.000 | 0.000 |
| 124 | 0.000 | 0.000 | 0.000 | 0.000 | 0.000 | 0.000 | 0.328 | 0.069 |
| BFRO001 |  |  |  |  |  |  |  |  |
| 202 | 0.444 | 0.429 | 1.000 | 1.000 | 0.000 | 0.000 | 1.000 | 1.000 |
| 204 | 0.000 | 0.000 | 0.000 | 0.000 | 1.000 | 1.000 | 0.000 | 0.000 |
| 212 | 0.556 | 0.571 | 0.000 | 0.000 | 0.000 | 0.000 | 0.000 | 0.000 |
